# Huanglongbing (Citrus Greening) Detection Using Visible, Near Infrared and Thermal Imaging Techniques

**DOI:** 10.3390/s130202117

**Published:** 2013-02-06

**Authors:** Sindhuja Sankaran, Joe Mari Maja, Sherrie Buchanon, Reza Ehsani

**Affiliations:** Citrus Research and Education Center, IFAS, University of Florida, 700 Experiment Station Road, Lake Alfred, FL 33850, USA; E-Mails: sindhu@ufl.edu (S.S.); jmaja@ufl.edu (J.M.M.); buchanon@ufl.edu (S.B.)

**Keywords:** citrus disease, visible-near infrared imaging, thermal imaging, support vector machine

## Abstract

This study demonstrates the applicability of visible-near infrared and thermal imaging for detection of Huanglongbing (HLB) disease in citrus trees. Visible-near infrared (440–900 nm) and thermal infrared spectral reflectance data were collected from individual healthy and HLB-infected trees. Data analysis revealed that the average reflectance values of the healthy trees in the visible region were lower than those in the near infrared region, while the opposite was the case for HLB-infected trees. Moreover, 560 nm, 710 nm, and thermal band showed maximum class separability between healthy and HLB-infected groups among the evaluated visible-infrared bands. Similarly, analysis of several vegetation indices indicated that the normalized difference vegetation index (NDVI), Vogelmann red-edge index (VOG) and modified red-edge simple ratio (mSR) demonstrated good class separability between the two groups. Classification studies using average spectral reflectance values from the visible, near infrared, and thermal bands (13 spectral features) as input features indicated that an average overall classification accuracy of about 87%, with 89% specificity and 85% sensitivity could be achieved with classification models such as support vector machine for trees with symptomatic leaves.

## 1. Introduction

In recent years, many invasive citrus diseases have spread to different parts of Florida affecting the citrus industry. Huanglongbing (HLB) or citrus greening is one of most serious citrus diseases resulting in an estimated loss of about $3 billion in terms of annual production value [1]. HLB has destroyed about 1 million commercial citrus trees in Florida. With about 550,000 acres of citrus orchards, it is challenging to scout (visually inspect) for HLB in the entire production area. Moreover, rapid spread of HLB coupled with increasing demand and costs for labor dictates the need for advanced sensing technologies for HLB detection in citrus. Remote sensing tools could accelerate the identification of HLB disease and application of appropriate control measures.

Remote sensing is emerging as an important component of precision agriculture for its ability to identify and define crop health. Changes in spectral reflectance can indicate physiological stress in trees that result from the changes in photosynthetic pigments such as chlorophyll, carotenoids and other factors. With improvements in spatial, spectral and temporal resolution of remote sensing, multispectral imagery remains advantageous due to its real-time or near real-time imagery for visual assessment [2–4].

Multispectral and thermal imaging systems have been used for stress detection in different crops [5–10]. Kriston-Vizi *et al.* [11] used multispectral imaging for water stress detection in mandarin and peach. A moderate relationship (R^2^ = 0.51–0.53) was found between the green (580–680 nm) and red leaf reflectance (490–580 nm) data with leaf water potential in mandarins. Similarly, Dammer *et al.* [9] used multispectral computer vision to determine head blight disease in winter wheat.

Our previous work involved the application of visible-near infrared spectra for HLB detection in citrus leaves [12–14]. These experiments were conducted in controlled (laboratory) and field conditions. The in-field data collection involved acquiring spectral data from the citrus leaves (healthy and HLB-infected symptomatic leaves) located on the side of the tree canopy. In these studies, we found that the spectral signature (350–2,500 nm) could be used to classify the HLB-infected trees from healthy ones with an accuracy of 90% and greater. The present study was motivated by the need for an optical sensor capable of detecting HLB from the top of the canopy, which otherwise cannot be accessed by the scouts. Moreover, imaging techniques are more robust as they provide a spectral response of the tree canopy over a larger area than those of other spectroscopic methods [15]. In addition to multispectral sensors, thermal sensors have been used in several studies [16–19] for plant stress detection. In recent years, there has been a growing trend in adoption of small autonomous unmanned aerial vehicles (UAVs) for agricultural applications. A combination of a small UAV with multispectral and thermal imaging techniques can provide an efficient solution for crop scouting and can potentially be a key sensing tool for crop stress detection.

The long term goal of this study is to integrate multispectral and thermal imaging with a small multi-rotor UAV platform for canopy stress detection. The specific objective of this work was to evaluate the application of multispectral and thermal imaging for HLB detection in citrus trees using a mobile ground-based sensor platform. For this purpose, three cameras (two multispectral and one thermal) were used to acquire spectral images from the top of the healthy and HLB-infected citrus canopies. The multispectral cameras were comprised of blue, green, red, red-edge, and near infrared filters, while the thermal camera consisted of mid-infrared filters.

## 2. Materials and Methods

### 2.1. Sensors and Experimental Set-Up

Two multiple camera arrays (MCA, MIC-005, Tetracam Inc., Chatsworth, CA, USA) with six sensor channels each were used for collecting high-resolution images from different wavelengths (blue to near infrared regions of the electromagnetic spectra). The spectral bands used were 440, 480, 530, 560, 610, 660, 690, 710, 740, 810, 850, and 900 nm. The band width was 10 nm. The bands were selected based on preliminary evaluation and previous work [13], along with availability of spectral filters for six-band camera. The thermal uncooled camera (Tau 640, FLIR Systems Inc., Boston, MA, USA) was used for collecting images from the thermal infrared region of the electromagnetic spectra. The specifications of the cameras are summarized in Table 1. The pixel values (8 bit image) represent the reflectance of the tree canopy. The lowest pixel value (0 or black) indicates minimum reflectance and the highest pixel value (255 or white) indicates maximum reflectance.

**Figure 1.**
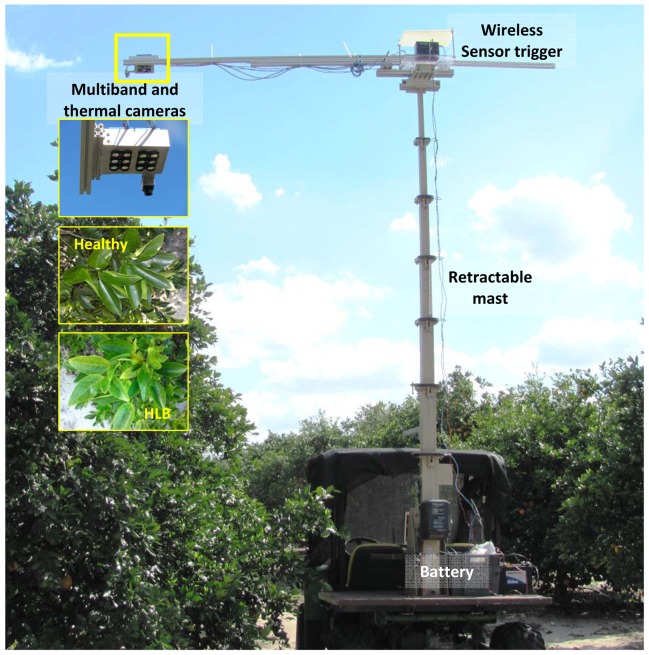
Experimental set-up showing the utility vehicle with sensors mounted on a retractable mast and representative tree bunch with healthy and HLB-infected leaves.

The experimental set-up (Figure 1) was comprised of two six-band cameras and a thermal camera mounted on a support platform placed on a telescoping mast (Floatograph Technologies, Silver Spring, MD, USA). The retractable mast was fixed to the back of an all-terrain utility vehicle (Gator, Deere and Company, Moline, IL, USA). The sensor support set-up was mounted such that the cameras covered the region of interest (top of citrus tree canopy). The six-band cameras were triggered wirelessly from a laptop computer; while thermal camera was connected via USB port during data collection. The six-band cameras were powered by a 12 V car battery; while the thermal camera received the power through the USB port of a laptop computer.

### 2.2. Data Collection

**Figure 2.**
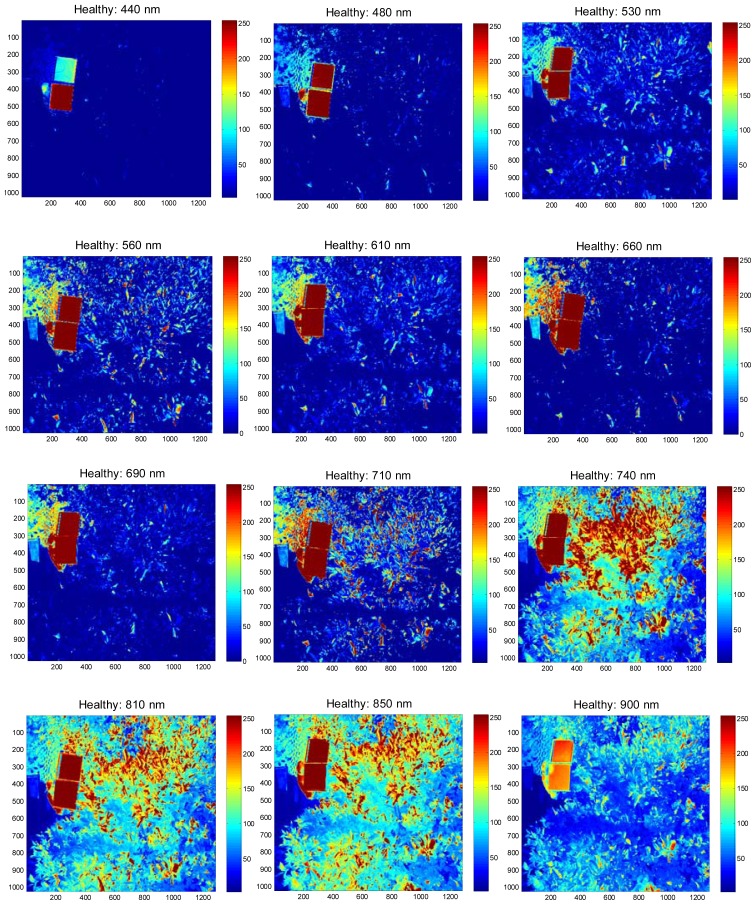
Pseudo color images that represent visible-near infrared spectral reflectance values of a healthy citrus tree. The field-of-view of the camera included the citrus tree canopy, the white reference panel (that appear as dark red squares in the images) and a small portion of soil (ground).

**Figure 3.**
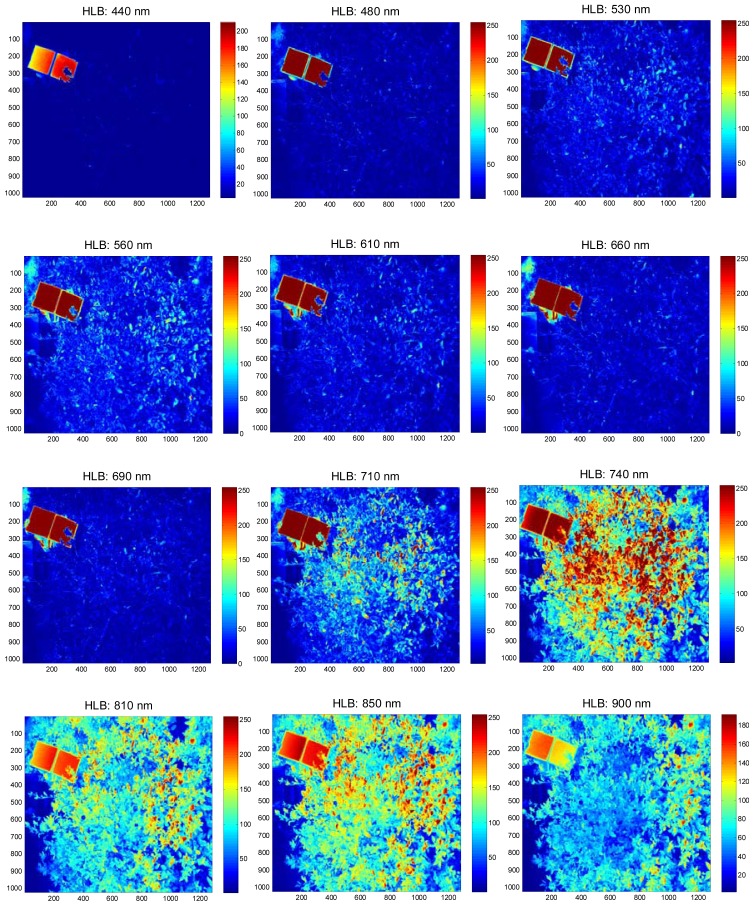
Pseudo color images that represent visible-near infrared spectral reflectance values of a HLB-infected citrus tree. The field-of-view of the camera included the citrus tree canopy, the white reference panel (that appear as dark red squares in the images) and a small portion of soil (ground).

**Figure 4.**
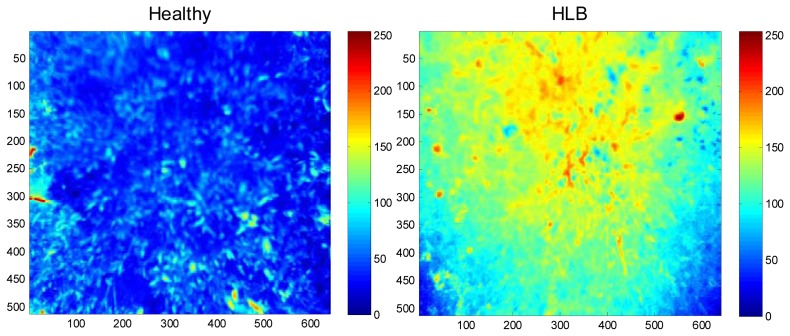
Pseudo color thermal images of a healthy and HLB-infected citrus tree. The field-of-view of the camera included the citrus tree canopy and a small portion of soil. The HLB-infected tree appears lighter than the healthy tree, due to higher temperatures.

The field experiments were carried-out at the University of Florida's Citrus Research and Education Center grove (Lake Alfred, FL, USA) during August and September of 2011. The data were collected between 11:00 a.m. and 3:00 p.m. and at a distance of 3 m above the tree canopy under natural light conditions (sunlight). During image acquisition, a white reflectance reference panel of 25.4 × 25.4 cm (Spectralon Reflectance Target, CSTM-SRT-99-100, Spectra Vista Corporation, Poughkeepsie, NY, USA) was used. The white reference panel in the images was used to perform corrections for variations in the light intensity. The spectral reflectance values of a tree canopy for each band will depend on the physiological status of the tree and its interactions with the light source. The field data were taken from 36 healthy and 38 HLB-infected trees (Valencia oranges). The HLB-infected trees had a few symptomatic leaves (chlorosis and blotchy mottle), but not completely covered with such leaves. Figures 2 and 3 illustrate representative pseudo color images in the visible-near infrared region from a healthy and HLB-infected tree, respectively. The pseudo colored images were developed from the digital values that indicate spectral reflectance using the Matlab image processing toolbox (ver. 7.6, The MathWorks Inc., Natick, MA, USA) with high reflectance areas appearing as dark red and low reflectance areas appearing as dark blue. Figure 4 shows representative pseudo colored thermal images of the tree canopies.

### 2.3. Image Preprocessing

During image preprocessing, each of the pixel values of individual multispectral images (12 bands) was corrected with the average pixel value (30 × 30 pixels) of the white reference panel in that image for calibration. After the correction, the central image region covering the canopy was averaged to compute the average pixel values for each of the multispectral image (about 300 × 300 pixels) using an image processing and analysis software, ImageJ (http://imagej.nih.gov/ij/; U.S. National Institutes of Health, Bethesda, MD, USA). A window size of 300 × 300 pixels was selected to avoid spectral signatures from other non-canopy features in the image (such as reference panel, soil, *etc.*). Similar procedure was followed for thermal images, where average canopy reflectance representing the canopy temperature was averaged. These pixel values representing the reflectance in the visible, near infrared and thermal infrared regions of the electromagnetic spectra were further used for data analysis.

### 2.4. Vegetation Indices

In addition to the average spectral reflectance value of each spectral band in an image, selected vegetation indices were computed that represent the physiological stress in plants. The vegetation indices computed were: structure insensitive pigment index (SIPI; 810 nm, 690 nm, 440 nm), Vogelmann red-edge index (VOG; 740 nm, 710 nm), modified red-edge normalized difference vegetation index (mNDVI; 740 nm, 690 nm, 440 nm), modified red-edge simple ratio (mSR; 740 nm, 710 nm, 440 nm), red-edge normalized difference vegetation index (RE-NDVI; 740 nm, 690 nm), simple ratio index (SR), and normalized difference vegetation index (NDVI) [20–25]. The NDVI and SR were computed using three different combinations with (810 nm, 660 nm), (850 nm, 660 nm), and (900 nm, 660 nm), and labeled as NDVI1, NDVI2 and NDVI3 or SR1, SR2 and SR3, respectively. These vegetation indices were calculated for each individual multispectral image.

### 2.5. Data Analysis

**Figure 5.**
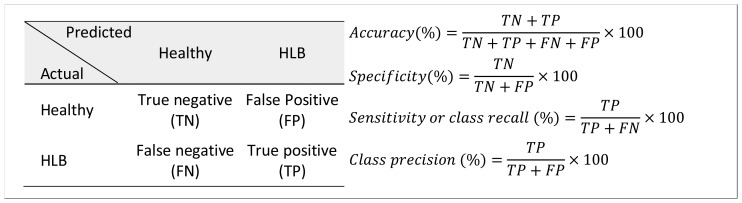
Confusion matrix and associated classification measures.

The Matlab program was used for data analysis. The summary statistics of spectral bands and vegetation indices for healthy and HLB-infected citrus trees were calculated. In addition, classification studies were performed to determine the effectiveness of the spectral features (average spectral reflectance values in visible, near- and thermal infrared bands) in categorizing the healthy and HLB-infected citrus canopies. The classification studies were performed using the ‘Statistics’ and ‘Bioinformatics’ toolbox of Matlab. The average spectral reflectance values of 13 bands (12 visible-near infrared bands + 1 thermal band) were used as input features for classification. The two output classes used in the analysis were “healthy” and “HLB”. The data were randomly separated into training and test datasets such that 75% of the data was utilized for training the classifier model; while 25% of the data was used for testing the developed classifier model. The classifiers tested were linear discriminant analysis (LDA), quadratic discriminant analysis (QDA), bagged decision tree (BDT) and support vector machine (SVM). The binary classification performance was assessed based on number of statistical measures such as accuracy, specificity, sensitivity (or class recall) and class precision, calculated from the confusion matrices (Figure 5). The specificity and sensitivity refer to healthy and HLB class classification accuracy, respectively. The data were randomized three times and the average classification measures were computed for each classifier model.

In addition to the classification performance, the three most significant spectral features and vegetation indices were identified based on the class separability criteria using Bioinformatics toolbox in Matlab. The class separability was evaluated based on two-sample t-tests (unpaired) with pooled variance estimate.

## 3. Results and Discussion

### 3.1. Spectral Bands and Vegetation Indices

**Figure 6.**
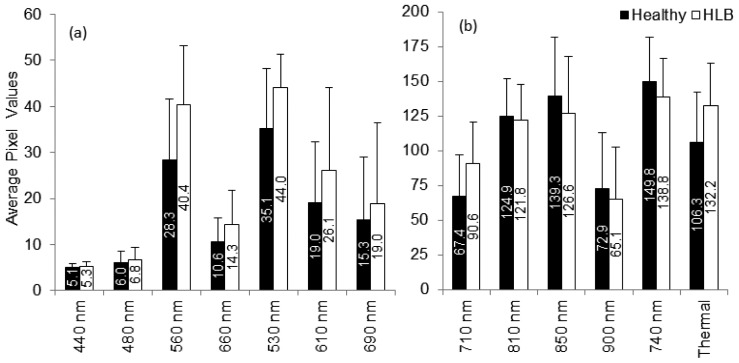
Summary statistics of individual spectral bands computed for healthy and HLB-infected trees. (**a**) Summary statistics of bands 440–690 nm, and (**b**) Summary statistics of bands from 710 nm and beyond.

The summary statistics with average and standard deviation of pixel values that represent reflectance from the canopy were computed for individual spectral bands. Figure 6 summarizes the results. It was observed that the average reflectance of the HLB-infected trees were higher than the healthy ones in the visible region of the electromagnetic spectra, whereas the average reflectance values of the healthy trees in the red-edge and near-infrared region of electromagnetic spectra was higher than those of HLB-infected trees. The healthy vegetation is known to have a good absorption in visible region, while exhibiting high reflectance in the near infrared region. This is due to the presence of leaf pigments such as xanthophylls, chlorophylls and carotenoids, which strongly absorb the visible region with little to no absorption in the near infrared region [26]. However, the water absorption bands along with other compounds are present in the mid-infrared (include thermal infrared) region of the electromagnetic spectra [26,27]. Due to the variations in visible-near infrared spectral reflectance between healthy and unhealthy canopies, several researchers have suggested the application of visible-near infrared spectroscopy for diagnosing stress conditions in different crops [27–33]. Similarly, thermal imaging can be used to detect plant stress [34,35].

The plant stress detection using thermal imaging is potentially possible due to the stomatal opening or other physiological changes that occur as a result of plant response/resistance to pathogens [36]. From Figure 6, it can be observed that the average spectral reflectance in the thermal infrared region was higher in HLB-infected canopies than that of healthy canopies. This indicated that the average temperature of HLB-infected tree canopies was higher than the healthy trees. When the spectral reflectance features were ranked using statistical methods, it was found that the three most prominent bands were 560 nm (green), 710 nm (far-red), and thermal. Given the symptoms of HLB (chlorosis), these bands could indicate stress. The band that exhibited minimal class separability was 810 nm. As the plant canopies reflect poorly in the near infrared region, the bands alone may not produce a good separability between healthy and HLB-infected classes. However, these bands may be used with other visible bands for better class separability.

**Figure 7.**
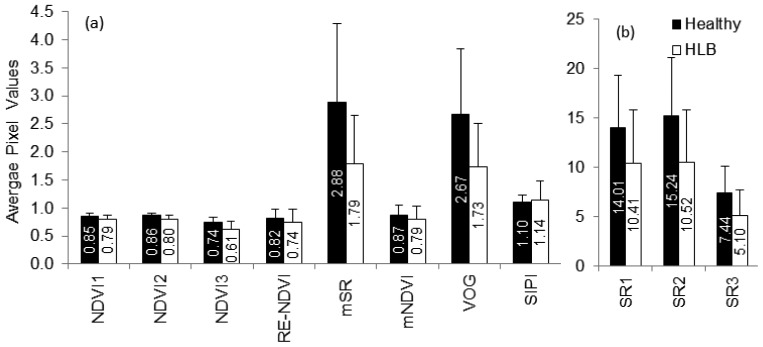
Summary statistics of vegetation indices computed for healthy and HLB-infected trees. (**a**) NDVI, RE-NDVI, mSR, mNDVI, VOG and SIPI values, and (**b**) SR values.

Vegetation indices have also been used to detect stress in plants [37–39]. Some of the indices indicating broadband greenness (NDVI, SR), narrowband greenness (RE-NDVI, mNDVI, mSR, VOG) and light-use efficiency (SIPI) were evaluated in this study. While NDVI and SR commonly indicate chlorophyll absorption by green vegetation, the other indices can be used to monitor plant stress through remote sensing. Figure 7 summarizes the average vegetation indices of healthy and HLB-infected canopies. The average values of all vegetation indices, except SIPI, were lower for HLB-infected canopies than for those of healthy canopies. The SIPI is a ratio of carotenoids to chlorophyll and is less sensitive to canopy structure variations [21]. Increasing carotenoid content relative to chlorophyll is an indication of plant stress [27,40]. Thus, it can be expected that HLB-infected trees will have higher average SIPI values than the healthy trees. When the separability of vegetation indices were tested, it was found that the NDVI, VOG and mSR exhibited maximum class separability, while, SIPI and mNDVI demonstrated low separability.

### 3.2. Classification Results

The classification studies were performed with reflectance values of visible, near infrared, and thermal bands as input features. Table 2 summarizes the classification results using the four classifiers. Among the classifiers, SVM yielded the maximum average classification accuracy with higher than 85% specificity and sensitivity. The LDA, QDA and BDT resulted in an overall average classification accuracy of about 80%, although there were variations in their specificity and sensitivity values. When the precision was predicted, it was found that all the classifiers performed well with 82% and higher values. When it comes to plant disease detection, it is important to have low false negatives (HLB classified as healthy) or higher sensitivity. The QDA and SVM generated the lowest false negatives (Table 2). The support vector machine is commonly used in remote sensing applications [41,42]. The major advantages of SVM over other methods include self-adaptation, limited need for large dataset for data training and good learning pace yielding good classification accuracy [42]. In this study, SVM resulted in a better classification performance than other classifiers, with an average overall accuracy of about 87%. This shows the potential of spectroscopic technologies based on SVM for disease detection in citrus. Berni *et al.* [33] used similar thermal and multispectral imaging sensors from unmanned aerial platforms for stress detection in different crops. They found that there was a good relationship between the leaf area index and NDVI in corn and olive (r^2^ = 0.5, 0.88) plants. Thus, the visible-near spectroscopy and thermal imaging have good prospects for remote sensing applications in agriculture.

## 4. Conclusions

Visible-near infrared and thermal imaging techniques were evaluated for detecting HLB-infected citrus trees in citrus orchards. The selection of spectral features is a critical step in identifying diseases. In this study, we evaluated the selected features to verify whether it was possible to identify HLB-infected trees with only a few spectral bands. The assessment of spectral reflectance values of visible and near infrared bands indicated that the HLB-infected trees reflected higher in the visible regions of spectra than in near infrared regions compared to healthy trees. In addition, wavebands such as 560 nm and 710 nm had good separability in visible-near infrared spectral regions. HLB symptoms can affect the greenness of the leaves (560 nm), which could be the cause for high separability at 560 nm. Similarly, red-edge (710 nm) is known to reflect physiological stress in plants [43].

The canopy temperature changes can also be used to indicate stress [16–19] as measured by the spectral reflectance in the thermal infrared region. It is important to develop the right analysis protocol, in addition to spectral band selection and sensor system development. For these reasons, classification studies were performed as a part of data analysis. During classification, the spectral reflectance values of 13 bands were used as input features to test various classifiers. Support vector machine resulted in an average overall classification accuracy of 87% with a minimum number of false negatives.

This study demonstrates the application of visible-near infrared with thermal imaging for remote sensing applications, which can also be automated. This is important technology for citrus orchards, as there is a need for frequent monitoring of diseases. Moreover, the sensing technology can be further expanded to larger agricultural area using aerial sensing platforms. One such sensing platform could be an UAV that can be used for high-resolution, low-altitude, aerial imaging. Our future studies will involve assessing the visible-near infrared imaging technologies for aerial sensing of HLB in citrus orchards using a small UAV system.

## Figures and Tables

**Table 1.** Sensor specifications used for data collection.

**Specifications**	**Multiband camera**	**Thermal camera**
Image resolution	1280 × 1024 (1.3 MP)	640 × 512
Lens	8.5 mm	13 mm
Image storage	8 bit	8 bit
Dimensions	203 mm × 179 mm × 101 mm	44.5 mm × 44.5 mm × 30.0 mm
Weight	1.55 kg	72 g
Wavelength	Six/camera (440–900 nm)	7.5–13.5 μm
Input voltage	12–14 VDC	4–6 VDC
Model	MCA, Tetracam Inc.	Tau 640, FLIR Systems Inc.

**Table 2.** Classification accuracy, specificity and sensitivity obtained from different classifiers.

**Classification results**	**LDA**	**QDA**	**BDT**	**SVM**
Accuracy (%)	81 ± 6	80 ± 6	80 ± 3	87 ± 3
Specificity (%)	85 ± 17	78 ± 22	93 ± 6	89 ± 11
Sensitivity or class recall (%)	78 ± 19	81 ± 13	67 ± 11	85 ± 17
Class precision (%)	87 ± 14	82 ± 17	91 ± 8	90 ± 9

## References

[1] Economic Impacts of Citrus Greening (HLB) in Florida, 2006/07–2010/11. http://edis.ifas.ufl.edu/fe903.

[2] Everitt J.H., Escobar D.E. The Status of Video Systems for Remote Sensing Applications.

[3] Everitt J.H., Escobar D.E., Cavazos I., Noriega J.R., Davis M.R. (1995). A three-camera multispectral digital video imaging system. Remote Sens. Environ..

[4] Mausel P.W., Everitt J.H., Escobar D.E., King D.J. (1992). Airborne videography: Current status and future perspectives. Photogramm. Eng. Remote Sens..

[5] Qin Z., Zhang M. (2005). Detection of rice sheath blight for in-season disease management using multispectral remote sensing. Int. J. Appl. Earth Obs. Geoinf..

[6] Ondimu S., Murase H. (2008). Water stress detection in sunagoke moss (rhacomitrium. canescens) using combined thermal infrared and visible light imaging techniques. Biosyst. Eng..

[7] Yang C., Everitt J.H., Fernandez C.J. (2010). Comparison of airborne multispectral and hyperspectral imagery for mapping cotton root rot. Biosyst. Eng..

[8] Cui D., Zhang Q., Li M., Hartman G.L., Zhao Y. (2010). Image processing methods for quantitatively detecting soybean rust from multispectral images. Biosyst. Eng..

[9] Dammer K.H., MÖller B., Rodemann B., Heppner D. (2011). Detection of head blight (Fusarium ssp.) in winter wheat by color and multispectral image analyses. Crop Prot..

[10] Bauriegel E., Giebel A., Geyer M., Schmidt U., Herppich W.B. (2011). Early detection of Fusarium infection in wheat using hyper-spectral imaging. Comput. Electron. Agric..

[11] Kriston-Vizi J., Umeda M., Miyamoto K. (2008). Assessment of the water status of mandarin and peach canopies using visible multispectral imagery. Biosyst. Eng..

[12] Sankaran S., Mishra A., Maja J.M., Ehsani R. (2011). Visible-near infrared spectroscopy for detection of Huanglongbing in citrus orchards. Comput. Electron. Agric..

[13] Sankaran S., Ehsani R. (2011). Visible-near infrared spectroscopy based citrus greening detection: evaluation of spectral feature extraction techniques. Crop Prot..

[14] Mishra A.R., Karimi D., Ehsani R., Lee W.S. (2012). Identification of citrus greening (HLB) using a Vis-NIR spectroscopy technique. Trans. ASABE.

[15] Sankaran S., Mishra A., Ehsani R., Davis C. (2010). A review of advanced techniques for detecting plant diseases. Comput. Electron. Agric..

[16] Chaerle L., Van Der Straeten D. (2000). Imaging techniques and the early detection of plant stress. Trends Plant. Sci..

[17] Chaerle L., Hulsen K., Hermans C., Strasser R.J., Valcke R., Höfte M., Van Der Straeten D. (2003). Robotized time-lapse imaging to assess in-plant uptake of phenylurea herbicides and their microbial degradation. Physiol. Plant..

[18] Jones H.G. (2004). Application of thermal imaging and infrared sensing in plant physiology and ecophysiology. Adv. Bot. Res..

[19] Oerke E.C., Steiner U., Dehne H.W., Lindenthal M. (2006). Thermal imaging of cucumber leaves affected by downy mildew and environmental conditions. J. Exp. Bot..

[20] Datt B. (1999). A new reflectance index for remote sensing of chlorophyll content in higher plants: Tests using eucalyptus leaves. J. Plant. Physiol..

[21] Penuelas J., Baret F., Filella I. (1995). Semi-empirical indices to assess carotenoids/chlorophyll *a* ratio from leaf spectral reflectance. Photosynthetica.

[22] Rouse J.W., Haas R.H., Schell J.A., Deering D.W. Monitoring Vegetation Systems in the Great Plains with ERTS.

[23] Sellers P.J. (1985). Canopy reflectance, photosynthesis and transpiration. Int. J. Remote Sens..

[24] Sims D.A., Gamon J.A. (2002). Relationships between leaf pigment content and spectral reflectance across a wide range of species, leaf structures and developmental stages. Remote Sens. Environ..

[25] Vogelmann J.E., Rock B.N., Moss D.M. (1993). Red edge spectral measurements from sugar maple leaves. Int. J. Remote Sens..

[26] Knipling E.B. (1970). Physical and physiological basis for the reflectance of visible and near-infrared radiation from vegetation. Remote Sens. Environ..

[27] Penuelas J., Filella I. (1998). Visible and near-infrared reflectance techniques for diagnosing plant physiological status. Trends Plant. Sci..

[28] Carter G.A. (1993). Responses of leaf spectral reflectance to plant stress. Am. J. Bot..

[29] Carter G.A., Cibula W.G., Miller R.L. (1996). Narrow-band reflectance imagery compared with thermal imagery for early detection of plant stress. J. Plant. Physiol..

[30] Price J.C. (1992). Estimating vegetation amount from visible and near infrared reflectance. Remote Sens. Environ..

[31] Krumov A., Nikolova A., Vassilev V., Vassilev N. (2008). Assessment of plant vitality detection through fluorescence and reflectance imagery. Adv. Space Res..

[32] Liu M., Liu X., Ding W., Wu L. (2011). Monitoring stress levels on rice with heavy metal pollution from hyperspectral reflectance data using wavelet-fractal analysis. Int. J. Appl. Earth Obs. Geoinf..

[33] Berni J.A.J., Zarco-Tejada P.J., Suarez L., Gonzalez-Dugo V., Fereres E. (2009). Remote sensing of vegetation from UAV platforms using lightweight multispectral and thermal imaging sensors. Int. Arch. Photogramm. Remote Sens. Spatial Inform. Sci..

[34] Chaerle L., Hagenbeek D., Bruyne E.D., Valcke R., Straeten D.V.D. (2004). Thermal and chlorophyll-fluorescence imaging distinguish plant-pathogen interactions at an early stage. Plant Cell Physiol..

[35] Vandivambal R., Jayas D.S. (2011). Applications of thermal imaging in agriculture and food industry-a review. Food Bioprocess Tech..

[36] Chaerle L., Leinonen I., Jones H.G., Straeten D.V.D. (2007). Monitoring and screening plant populations with combined thermal and chlorophyll fluorescence imaging. J. Exp. Biol..

[37] Carter G.A., Miller L.M. (1994). Early detection of plant stress by digital imaging within narrow stress-sensitive wavebands. Remote Sens. Environ..

[38] Jackson R.D., Slater P.N., Pinter P.J. (1983). Discrimination of growth and water stress in wheat by various vegetation indices through clear and turbid atmospheres. Remote Sens. Environ..

[39] Lichtenthaler H.K., Wenzel O., Buschmann C., Gitelson A. (1998). Plant stress detection by reflectance and fluorescence. Ann. N. Y. Acad. Sci..

[40] Young A., Britton G., Alscher R.G., Cumming J.R. (1990). Carotenoids and Stress. Stress Responses in Plants: Adaptation and Acclimation Mechanisms.

[41] Rumpf T., Mahlein A.K., Steiner U., Oerke E.C., Dehne H.W., Plümer L. (2010). Early detection and classification of plant diseases with Support Vector Machines based on hyperspectral reflectance. Comput. Electron. Agric..

[42] Mountrakis G., Im J., Ogole C. (2011). Support vector machines in remote sensing: a review. ISPRS J. Photogramm. Remote Sens..

[43] Li X., Lee W.S., Li M., Ehsani R., Mishra A.R., Yang C., Mangan R.L. (2012). Spectral difference analysis and airborne imaging classification for citrus greening infected trees. Comput. Electron. Agric..

